# Immune cell infiltration and prognostic index in cervical cancer: insights from metabolism-related differential genes

**DOI:** 10.3389/fimmu.2024.1411132

**Published:** 2024-05-22

**Authors:** Boyi Ma, Chenlu Ren, Yadong Yin, Shuhua Zhao, Jia Li, Hong Yang

**Affiliations:** Department of Obstetrics and Gynecology, Xijing Hospital, Air Force Medical University, Shaanxi, Xi’an, China

**Keywords:** mitochondrion, energy metabolism, cervical cancer, immune, IscU

## Abstract

**Background:**

Cervical cancer remains a significant gynecologic malignancy in both China and the United States, posing a substantial threat to women’s lives and health due to its high morbidity and mortality rates. Altered energy metabolism and dysregulated mitochondrial function play crucial roles in the development, growth, metastasis, and recurrence of malignant tumors. In this study, we aimed to predict prognosis and assess efficacy of anti-tumor therapy in cervical cancer patients based on differential genes associated with mitochondrial metabolism.

**Methods:**

Transcriptomic data and clinical profiles of cervical cancer patients were retrieved from the TCGA and GEO databases. Differential gene-related cellular pathways were identified through GO, KEGG, and GSEA analyses. Prognostic indices were constructed using LASSO regression analysis. Immune cell infiltration was assessed using CIBERSORT and ssGSEA, and the correlation between immune checkpoint inhibitor genes and differential genes was examined. Tumor mutation load (TMB) and its association with prognostic indices were analyzed using nucleotide variant data from the TCGA database. Patient response to immunotherapy and sensitivity to antitumor drugs were determined using the TIDE algorithm and the oncoPredic algorithm, respectively.

**Results:**

A prognostic index based on metabolism-related differential genes was developed to predict the clinical outcome of cervical cancer patients, enabling their classification into two distinct subtypes. The prognostic index emerged as an independent risk factor for unfavorable prognosis. The high-index group exhibited a significantly worse overall prognosis, along with elevated tumor mutation burden (TMB), increased immune cell infiltration, and lower TIDE scores, indicating a potential benefit from immunotherapy. Conversely, the low-index group demonstrated increased sensitivity to metabolism-related antitumor agents, specifically multikinase inhibitors.

**Conclusion:**

The aim of this study was to develop a prognostic index based on differential genes associated with mitochondrial metabolism, which could be used to predict cervical cancer patients’ prognoses. When combined with TIDE and TMB analyses, this prognostic index offers insights into the immune cell infiltration landscape, as well as the potential efficacy of immunotherapy and targeted therapy. Our analysis suggests that the Iron-Sulfur Cluster Assembly Enzyme (ISCU) gene holds promise as a biomarker for cervical cancer immunotherapy.

## Introduction

The latest 2023 findings from the American Cancer Society indicate that cervical cancer continues to be the primary gynecological cancer in the United States, presenting a major risk to women’s well-being and survival (1). Mitochondria are essential in the progression, spread, and reappearance of cancerous growths by affecting energy production and proliferation (2). Metabolic reprogramming in cervical cancer drives malignant behaviors such as metastasis and progression, with structural and functional changes in mitochondria influencing tumor behavior (3). Thus, cell metabolic reprogramming, specifically alterations in mitochondrial metabolism, impacts the progression of cervical cancer and can be targeted for tumor therapy.

Mitochondria, vital cell components, play a key role in various biological processes including cellular metabolism, production of reactive oxygen species (ROS), and programmed cell death. Various human diseases, including neurodegenerative disorders and cancer (4, 5), have been associated with dysfunction in mitochondria. Cancer is characterized by metabolic reprogramming (6), which can be caused by changes in mitochondrial metabolic pathways due to mutations in oncogenes and tumor suppressor genes (7). Research on the dynamics of mitochondria has shown that disruptions in the merging and splitting of mitochondria play a role in the development and advancement of various types of cancer, impacting the growth of cancer cells, their spread to other parts of the body, resistance to drugs, and the environment surrounding the tumor (8). Mitochondrial dysfunction triggers several mitochondrial retrograde signaling pathways through the release of molecules originating from mitochondria (such as ROS, calcium, oncometabolites, and exported mtDNA) trigger the activation of pathways that respond to mitochondrial stress (mtUPR and ISR), ultimately facilitating the advancement of tumors towards malignancy. The tumor microenvironment can be influenced by perturbations in energy metabolism or mitochondrial function, resulting in impaired anticancer function of immune cells (9, 10). The findings indicate that targeting mitochondrial retrograde signaling in cancer cells and modulating mitochondrial metabolism have promise as strategies for halting cancer progression to malignancy. In the context of cervical cancer, genetic alterations in mitochondrial energy metabolism result in abnormal changes in mitochondrial structure and function, thereby exerting an influence on cervical carcinogenesis and disease progression.

Immunotherapy is commonly acknowledged as a highly effective treatment option for cervical cancer, with a growing understanding of the promise of immune checkpoint inhibitors (11–13). Inhibitors of poly (adenosine diphosphate ribose) polymerase have the ability to trigger the checkpoints of programmed death 1 (PD-1) and programmed death ligand 1 (PD-L1), ultimately suppressing the growth of cervical cancer cells (14, 15). Immunotherapy is frequently used in conjunction with chemotherapy and radiation to improve and speed up treatment results. Besides immune checkpoint inhibitors, there is another treatment choice available in the form of chimeric antigen receptor-engineered (CAR) immune cells (16). While immunotherapy has shown promise in clinical settings, its therapeutic efficacy varies significantly on an individualized basis, underscoring the ongoing need for exploring novel technological approaches to improve its effectiveness.

This research involved a thorough bioinformatics examination utilizing the TCGA repository to explore the expression patterns, genetic alterations, biological roles, and connections with immune cells and immune functions of distinct genes associated with mitochondrial metabolism in cervical cancer. Additionally, we used these distinct genes to create a prognostic score to forecast the survival results of individuals with cervical cancer. Patients with cervical cancer were categorized into two separate clinical subgroups according to their prognostic score, revealing notable variations in response to immunotherapy and sensitivity to medications. Our analysis also revealed the potential key role of ISCU genes in regulating immunotherapy efficacy and tumor progression.

## Materials and methods

### Data collection

Cervical cancer transcriptomic data, mutation data, and clinical data were retrieved from the TCGA and GEO databases. A total of 299 samples from the TCGA database were included for the analysis of differential gene expression, with matched clinical information. For the construction of the prognostic index, 283 cervical cancer samples were used (16 samples without clinical staging were excluded). The validation set comprised 55 cervical cancer samples from the GSE52904 dataset in the GEO database. Samples lacking matched clinical information in both the TCGA and GEO databases were excluded from the analysis.

### Differential gene expression and gene mutation analysis

Mitochondrial energy metabolism-related genes differentially expressed by DESeq2 were downloaded from the Human.MitoCarta3.0 database. The TCGA data was processed using the easyTCGA R package, and the mutation data was analyzed using the maftools R package to visually assess the mutation frequency of genes in patients. The FDR value ≤ 0.05 was used as the differential gene screening criterion. Mitochondrial metabolism-related differential genes were obtained by intersecting genes obtained by Human.MitoCarta3.0 with differential genes.

### Gene enrichment analysis

Differential gene enrichment analysis was conducted with the R package clusterProfiler to explore the variance in gene enrichment between healthy cervix and cervical cancer. A statistically significant P-value threshold of 0.05 was applied to identify gene sets with less than or equal to 5 genes, and those with more than 5,000 genes.

### Prognostic index construction

The differential genes related to mitochondrial metabolism were selected for 10-fold cross-validation using Lasso regression. The lambda value with the smallest mean error, determined from the cross-validation results, was used to select the genes to be included in the prognostic index. The prognostic index was constructed by multiplying the included genes with their regression coefficients and summing them up. Following this, the individuals were categorized into groups with high and low indexes according to the average expression level. Survival analysis was conducted to assess the prognostic significance of the prognostic index. Additionally, the significance of the prognostic index was validated using the GEO database. Furthermore, multifactorial Prognostic indexes were investigated using Cox regression analysis for cervical cancer patients to confirm their independence as prognostic factors.

### Correlation analysis of immune cell infiltration and immune function

Utilizing the R software CIBERSORT, we conducted an examination of the relationship between immune cell penetration and immune activity. This assessment enabled us to ascertain the proportion of 22 distinct immune cell categories in the TCGA dataset for individuals with normal health and those diagnosed with cervical cancer, in addition to those classified in high and low index categories. Additionally, we conducted Gene Set Enrichment Analysis using sets of genes related to the immune system to assess differences in immune functions among groups with high and low indices. Furthermore, we compared the activity of immune functions between the two risk groups. To evaluate the immune stroma and immune microenvironment, we calculated immune stroma and immune microenvironment scores using the R package ESTIMATE.

### Tumor mutation load analysis

This study analyzed the tumor mutation load (TMB) to evaluate the mutation rate of samples by utilizing mutation data from cervical cancer patients in the TCGA database. We specifically analyzed the mutation differences between the high index and low index groups. Additionally, we categorized individuals with cervical cancer into two cohorts according to the median TMB in order to assess the correlation between the predictive measure of mutation burden and its influence on immunotherapy within the high and low index categories.

### Immunotherapy response

The Tumor Immune Dysfunction and Exclusion (TIDE) platform was utilized in cervical cancer patients to forecast the response to immunotherapy directed at PD-1 and CTLA4. The TIDE prediction score was employed to evaluate the likelihood of response to immunotherapy, with a negative correlation indicating a poorer response. In order to delve deeper into the variations in immunotherapy response, we analyzed the TIDE scores of patients in both high-risk and low-risk categories.

### Prediction of drug sensitivity

Using the R package oncoPredict, drug sensitivity for cervical cancer patients in the TCGA database was predicted in this research. Each patient’s sensitivity score was determined by utilizing the calcPhenotype function. We evaluated the differences in drug sensitivity for various medications by comparing sensitivity scores of the high and low index groups.

### Survival analysis and clinical staging and protein level analysis

Survival analyses were conducted using the R package Survival. To investigate the differential gene expression changes across different clinical stages of cervical cancer, we obtained data from the GEPIA2 online website. Additionally, gene expression data was obtained through a search in the HPA database.

### Immunohistochemistry

IHC staining was conducted after tissue slides were deparaffinized in xylene and rehydrated in alcohol, followed by 3%H2O2 treatment to inactivate endogenous peroxidase. A microwave-based antigen repair procedure was then performed in 0.1M sodium citrate buffer at pH 6.0. After blocking sections with 5% normal goat serum for 30 minutes at room temperature, primary antibodies were then left to incubate overnight at 4°C. For nuclei visualization, poly-HRP secondary antibody was incubated for 60 minutes at room temperature in the dark, followed by counterstaining with hematoxylin. Our hospital’s pathologists analyzed the staining results from images acquired with an Caseviewer scanner. ISCU expression was scored based on staining intensity as follows: 0 (negative), 1 (weak), 2 (moderate), and 3 (strong). The positive proportion area ranged from 1 (0–25%) to 4 (76%-100%). Scores of 0–12 were defined as ISCU expression results. This research project has obtained approval from the appropriate ethics committee or institution and is being conducted in strict adherence to ethical guidelines. The study upholds the rights and privacy of participants, ensuring the confidentiality of their personal information (KY20222112-C-1).

Inclusion criteria: 1) Patients with cervical cancer who have not received any anti-tumor treatment prior to surgery; 2) All cervical cancer patients have been confirmed by pathological diagnosis, and normal cervical tissues have been confirmed by pathologists to be free of any benign or malignant tumors; 3) Complete clinical and pathological data are available. Pathological diagnoses have been confirmed by two or more senior pathologists.

Exclusion criteria: 1) Patients with a history of previous gynecological tumor surgery; 2) Patients with recurrent cervical squamous cell carcinoma; 3) Patients with concurrent infectious diseases, rheumatic autoimmune diseases, cardiovascular and cerebrovascular diseases, and other major malignant tumors.

### Statistical analysis

The data was analyzed using R version 4.2.2. Statistical differences between the two groups were compared using a two-tailed Student’s t-test. Survival analysis was conducted using the Kaplan-Meier estimate and log-rank test. Statistical significance was established with a p-value below 0.05 (p<0.05).

## Results

### Differential expression gene screening

Data from the TCGA database was collected for cervical cancer patients and normal cervical tissues, including transcriptomic and clinical information. By acquiring the distinct genes and comparing them with genes related to mitochondrial metabolism, a sum of 1,123 differential genes related to metabolism were discovered through the utilization of the DESeq2 software. Among these genes, 754 were up-regulated, and 369 were down-regulated. The expression of the differential genes was visualized in **Supplementary Figure S1A** using a volcano plot. Additionally, **Supplementary Figure S1C** illustrated the relationship between the 50 genes with the most significant differences and clinical traits. The analysis revealed that the metabolic alterations in cervical cancer were closely associated with HPV infection status, tumor pathology type, and clinical stage. These findings suggest that metabolic gene alterations may have implications for the prognosis of cervical cancer patients.

### Functional enrichment and mutation analysis of metabolism-related genes

**Figure 1:**
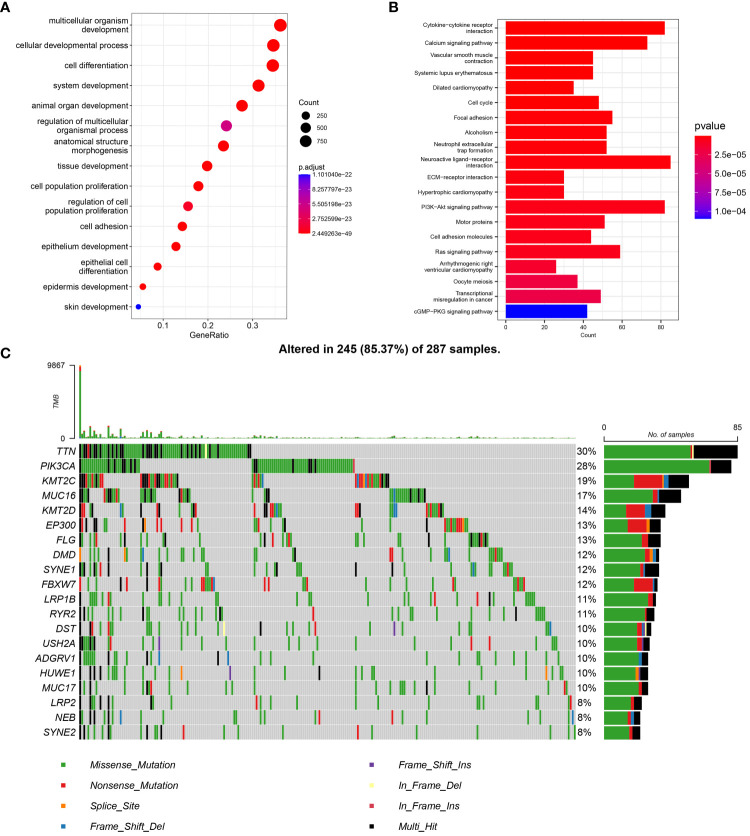
Metabolic differential gene expression, enrichment analysis and analysis with mutations. **(A)** Metabolic differential gene GO enrichment analysis in cervical cancer. **(B)** Differential gene KEGG enrichment analysis. **(C)** Mutation analysis of cervical cancer metabolic differential genes the top 20 genes with the most significant differences were selected.

The GO enrichment analysis showed that the differentially expressed genes were mainly enriched in the progression of cellular tissues and differentiation of cells. Cell structure enrichment was mainly seen in chromosomes, with molecular function enrichment in signal transduction and cell activation (**Figure 1A**). Analysis of KEGG enrichment revealed that the genes showing differences were primarily associated with interactions between cytokines and cells, binding of neural ligands to receptors, and the PI3K-Akt signaling pathway, among other pathways (**Figure 1B**).

To comprehensively explore the functions of the differential genes and avoid the exclusion of genes with insignificant differences by traditional enrichment algorithms, GSEA enrichment analysis was conducted. The analysis revealed the involvement of differential genes in cell cycle-related functions (**Supplementary Figure S1B**). Metabolism-related genes could potentially influence the outlook of individuals with cervical cancer, paving the way for the creation of a prognostic tool.

Mutation frequency analysis indicated that mitochondrial metabolism-related differential genes exhibited high mutation frequencies. The top 10 genes ranked by differences displayed mutation frequencies above 12%, while the mutation frequencies of the top 20 genes ranked by differences were above 8% (**Figure 1C**). Analyses of enrichment and mutation revealed that genetic mutations in metabolism-related genes could impact physiological functions like tumor metabolism, signal transduction, and tissue progression, ultimately affecting tumor progression, metastasis, and other biological processes.

### Metabolism-related gene prognostic index construction and survival analysis

Based on the aforementioned analysis, we propose a reasonable hypothesis that the survival time of cervical cancer patients can be predicted by constructing a metabolism-related gene index. To validate this hypothesis, we utilized a training set comprising 283 cases of TCGA cervical cancer patients with complete clinical information, and an external validation set consisting of 55 cases of cervical cancer from the GEO database (GSE52904) with complete clinical information. The prognostic index’s effectiveness was evaluated using the validation set.

**Figure 2:**
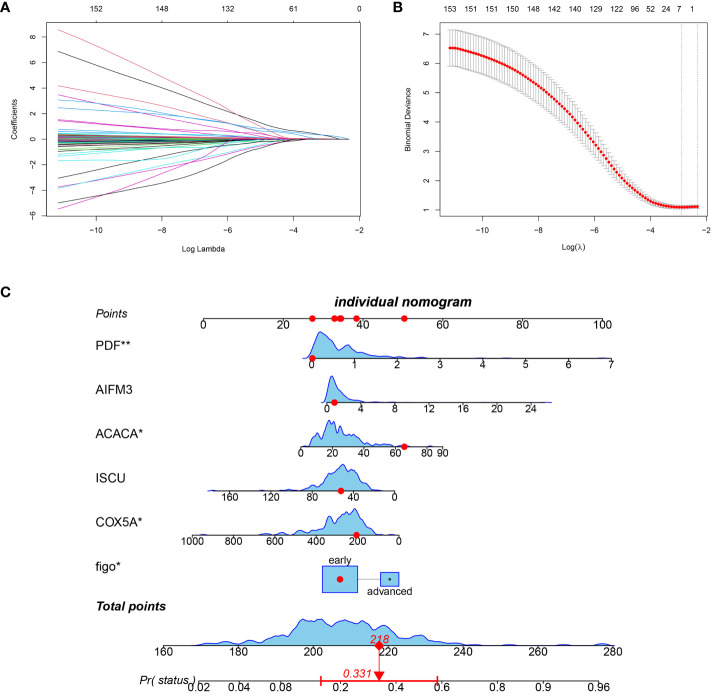
Prognostic score construction. **(A)** 10-fold cross-validation to adjust lasso regression model parameter selection. **(B)** Differential gene lasso regression analysis. **(C)** Nomogram column line graph visualization predicting patients’ prognostic index scores (in red, the prognostic score of one patient in the TCGA database is randomly displayed).

In order to enhance the clinical value of the index, clinical indicators were also taken into consideration during the construction of the prognostic index. A predictive score consisting of six factors was created using LASSO Cox regression analysis (**Figures 2A, B**). The prognostic index for every individual was forecasted by utilizing the equation established from the LASSO model: Prognostic Index = (Figo*0.205824) -(COX5A*0.000026) -(ISCU *0.000331) + (ACACA*0.003564) + (AIFM3*0.024294) + (PDF*0.273327). The predictive performance of the prognostic index was effectively demonstrated through column line graphs, with an AUC of 0.81 (**Figure 2C**). Evaluation metrics such as the Clinical Impact Curve, DCA Decision Curve, and Calibration Curve indicated that the prognostic model exhibited favorable performance (**Supplementary Figures S2B–D**). Single-gene survival analysis demonstrated significant differences in survival for all prognostic index genes, except for AIFM3 (**Supplementary Figure S3**).

**Figure 3:**
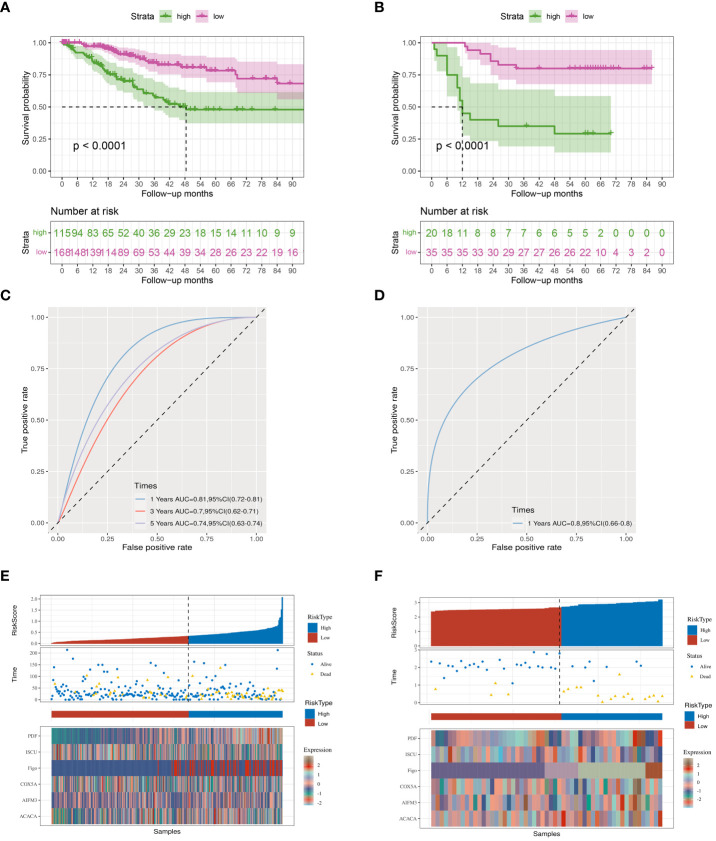
Kaplan-Meier curves. **(A)** TCGA database survival curves. **(B)** GEO database survival curves. **(C, D)** The AUC curve of the prognostic index model in the TCGA, GEO database. **(E, F)** Prognostic index grouping of samples in TCGA, GEO databases.

To further validate the prognostic index, A high index sample and a low index sample were selected from the training set. Survival rates were graphed, showing notable variances in survival rates between the groups with high and low indices (**Figure 3A**). This significant survival difference was also observed in the validation set (**Figure 3B**). Multivariate Cox regression analyses was performed to assess whether the prognostic index could function as an independent prognostic factor, revealing that the clinical data and prognostic index were indeed independent prognostic factors (P=0.045) (**Table 1**). In the training cohort, we observed that the prognostic index exhibited an AUC of 0.81, 0.70, and 0.74 at 1-, 3-, and 5-year intervals respectively (**Figure 3C**). While the validation set demonstrated an AUC value of 0.80 for a duration of one year (**Figure 3D**), it was not feasible to calculate AUC values for three and five years due to limited data collection time in this set. Furthermore, our findings indicate that the prognostic index effectively distinguishes between populations with varying survival outcomes, thereby reinforcing the significance of our research (**Figures 3E, F**). However, since the dataset with longer survival times lacked essential clinical information (such as FIGO), it could not fulfill our requirements; hence no AUC values were available for longer durations in the validation set.

**Table 1:** Multivariate analysis of clinical information and prognostic index of patients with cervical cancer.

Variable	P.value	Hazard.Ratio	X95.CI
Age	0.663	1.010	0.967–1.053
**Stage**	**0.017**	0.328	0.132–0.818
T	0.074	1.975	0.937–4.166
N	0.525	1.409	0.489–4.057
**Index**	**0.045**	0.154	0.025–0.959

Bold emphasis shows the results of analyses with statistical differences.

### Immunoassay of genes related to cervical cancer metabolism

Approval has been granted for the use of immunotherapy in treating late-stage cervical cancer, but there are still obstacles to overcome. These include determining the best timing for immunotherapy, choosing the right combination of drugs, and addressing resistance to immunotherapy. To address these issues, we investigated immunization-related aspects of cervical cancer.

Through immunological analyses of surviving and deceased cervical cancer patients, we observed significant differences in immune scores, immune microenvironment, and survival outcomes (**Supplementary Figures S4A–C**). These immune phenomena align with current studies highlighting the significant impact of immunotherapy on cervical cancer prognosis. Building upon these findings, we postulate a potential correlation between the mitochondrial metabolic prognostic index and immunotherapy in cervical cancer.

**Figure 4:**
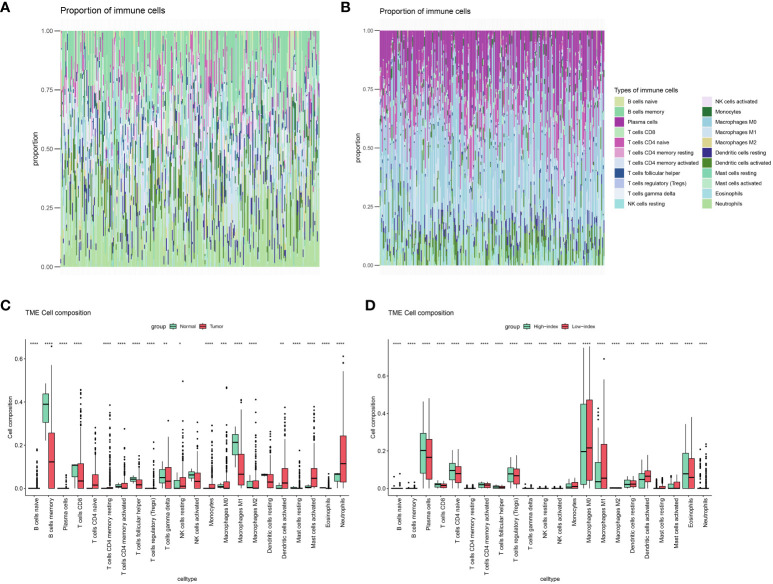
Immune landscape assessed by CIBERSORT algorithm. **(A)** Heatmap and **(C)** bar plot of abundance of 22 subtypes of immune cells in Normal versus cervical cancer patients. **(B)** Heatmap and **(D)** bar plot of abundance of 22 subtypes of immune cells in High index versus low index groups.(P values are shown as: *P < 0.05; **P < 0.01; ***P < 0.001; ****P < 0.0001).

Immunoinfiltration analysis demonstrated significant differences in memory B lymphocytes between normal samples and cervical cancer (**Figure 4A**), while the high and low index groups exhibited substantial disparities in plasma cells. Significant variations were noted between the high and low index groups in relation to immune cells linked to immunotherapy, such as plasma cells and M1 macrophages (**Figure 4B**), prompting speculation that the metabolic index may predict the effectiveness of immunotherapy in patients with cervical cancer.

Several immune cells from normal and cervical cancer samples, as well as from high- and low-index groups, were analyzed to test this hypothesis further. Surprisingly, the groups with high and low indexes showed notable variations in nearly all immune cells, exceeding the impacts seen in both the control and cervical cancer groups (**Figures 4C, D**). Furthermore, the analysis of immune processes revealed significant disparities not only in the proportions of immune cells but also in the underlying immune mechanisms, encompassing nearly all immune processes (**Supplementary Figure S5**). These findings provide compelling evidence supporting our initial suspicion that the metabolic difference index holds predictive value for the immunotherapeutic efficacy of cervical cancer patients.

### Prognostic index correlates with immunosuppressant genes, tumor mutation burden

**Figure 5:**
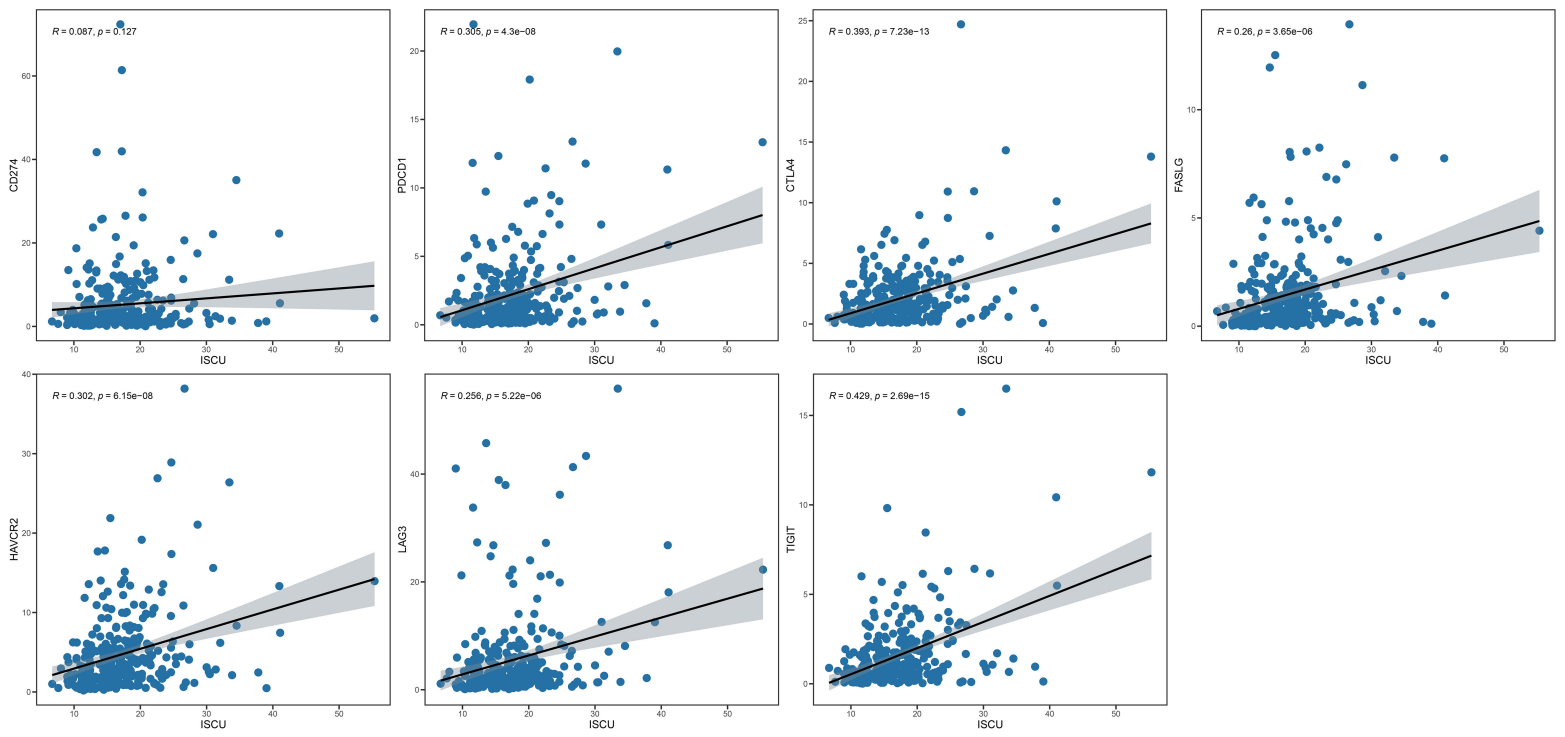
ISCU and immune checkpoint inhibitor genes (CD274, PDCD1, CTLA4, FASLG, HAVCR2, LAG3, TIGIT). ISCU, Iron-Sulfur Cluster Assembly Enzyme.

Immunoassays have shown that metabolic markers can predict immune cell levels and immune responses in individuals with cervical cancer. Immunosuppressive drugs have been widely used to treat advanced and recurrent cervical cancer, with the goal of determining if the prognostic index can impact outcomes through the immunosuppressive pathway. Gene correlation analysis revealed that among the prognostic index genes, only ISCU exhibited a significant correlation with immunosuppressant genes (**Figure 5**, **Supplementary Figures S6**, **S7**). Based on this finding, we hypothesize that ISCU may play a pivotal role in the immune-predictive capacity.

**Figure 6:**
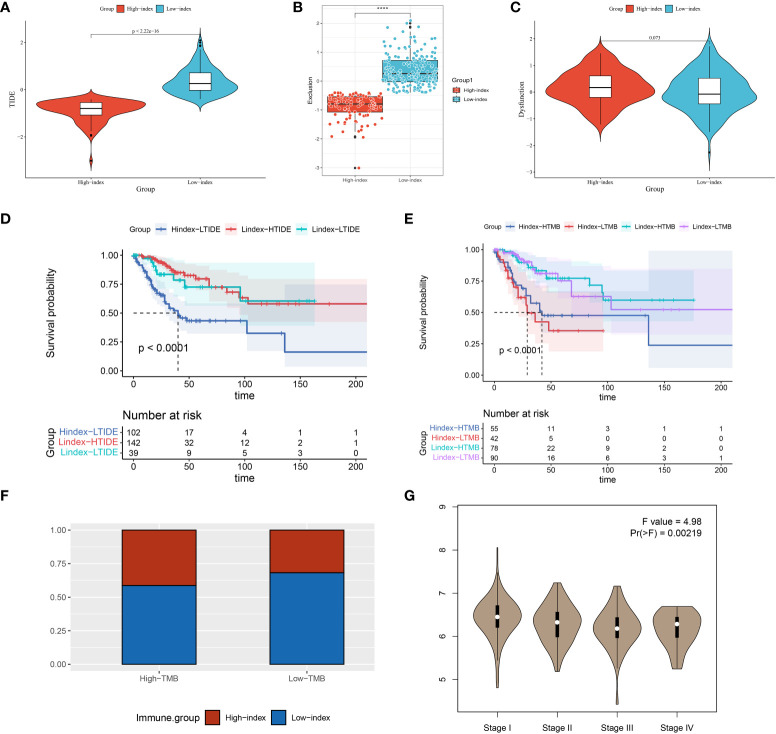
TIDE, KM, TMB analysis in high and low prognostic index groups and ISCU correlation with clinical staging. **(A–C)** A comparison of high and low index groups based on TIDE analysis and T-cell exclusion and dysfunction scores. With the Wilcoxon test, scores between the two subgroups of IRGPI were compared. ****P < 0.0001. **(D)** Analysis of survival between high and low index groups versus high and low TIDE scores; **(E)** The survival analysis compared the high and low index groups with the high and low TMB scores; **(F)** Comparison of high- and low-index groups versus high- and low- TMB groups; **(G)** Expression of ISCU in different stages of cervical cancer.

Tumor mutation load (TMB) is closely associated with immunosuppressant response, with a more pronounced effect of inhibitor treatment observed in cases with higher mutation loads. We sorted the samples into high and low mutation groups by determining the median mutation index of each sample in order to study the relationship between index genes and TMB. The findings revealed that 41.4% of the high mutation group exhibited a high index, whereas 68.2% of the low mutation group displayed a low index (**Figure 6F**). We grouped the samples into four categories and performed survival analysis based on the prognostic index and tumor mutational burden (TMB) results. The results of our study showed that patients with a low index and high mutational burden had the best prognosis, while those with a high index and low mutation burden had the worst outcome (see **Figure 6E**). These results further support our hypothesis that prognostic scores can serve as predictors of immunotherapy response in patients.

Our model’s predictive outcomes shed light on the reasons why certain patients with a high tumor burden experience poor immunotherapy efficacy. Furthermore, our results suggest that a significant proportion of patients in the low tumor load group have the potential to benefit from immunotherapy.

### TIDE analysis and drug sensitivity screening

Immunosuppressant therapy has demonstrated remarkable therapeutic efficacy in select patients with cervical cancer. The TIDE score is employed to assess the probability of tumor immune escape by analyzing the gene expression patterns in tumor specimens. To assess the predictive role of prognostic index-related genes in immunotherapy response, we conducted an analysis to examine the correlation between prognostic scores and TIDE scores.

The examination findings showed notable variances in TIDE rating and exclusion among the high and low index categories. Notably, the high index group exhibited a lower TIDE score, suggesting a potential for improved therapeutic efficacy with immune checkpoint inhibitors (**Figures 6A–C**). Patients were categorized into high TIDE and low TIDE groups using the median value obtained from the TIDE analysis. During the survival analysis, it was discovered that patients with a low predictive score and low TIDE had better results than those with a high score and low TIDE when examined collectively (all patients in the high score group were part of the low TIDE group). The findings suggest that the prognostic score may play a more significant role in forecasting the prognostic outcome (**Figure 6D**).

Additionally, we investigated the correlation between risk assessments and drug responsiveness. Drug sensitivity scores were derived from the gene expression data of cervical cancer patients using the calcPhenotype function within the oncoPredict package. The low-risk group showed more sensitivity to various chemotherapeutic agents, such as IRAK4, Dihydrorotenone, Gallibiscoquinazole, OF-1, JQ1, and more, when compared to the high-risk group (**Supplementary Figure S8**).

### Prognostic index genes and clinical staging of cervical cancer

We used the GEPIA database to examine the connection between prognostic index-associated genes and clinical stage. In advanced cervical cancers, ISCU expression was found to be decreased, whereas in early cervical cancers it was observed to be increased according to our analysis. Our observation prompted us to consider the possibility that the ISCU gene could have a protective function in the advancement of cervical cancer (**Figure 6G**, **Supplementary Figure S8**).

**Figure 7:**
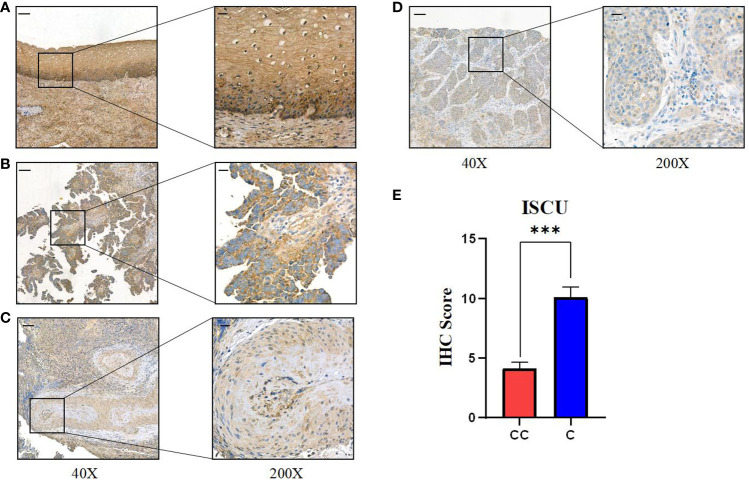
Expression of key gene ISCU in different stages of human cervical cancer tissues. **(A)** ISCU expression in normal cervical squamous epithelium; Expression of ISCU protein levels in patients with **(B)** stage I cervical cancer **(C)** stage II cervical cancer **(D)** stage III cervical cancer. **(E)** Quantitative results of ISCU expression in 40 cervical cancer patients and 10 control normal cervical tissues (40X: “-”100 micrometers; 200X: “-”20 micrometers). (P values are shown as: ***P < 0.001).

To evaluate the trustworthiness of the prognostic index model, 40 samples of cervical cancer tissues and 10 samples of normal cervical tissues (obtained from patients who had hysterectomy for non-malignant conditions) were acquired from the Department of Gynecology at XiJing Hospital. Immunohistochemical analysis showed a connection between ICSU protein expression and RNA levels, with the highest expression in healthy tissues decreasing as the stage advanced (**Figure 7**, **Table 2**). Cervical tissues were only available for patients with stages I-III, so the expression in stage IV patients remains unknown. Based on our analysis, we speculate that ISCU could be a useful indicator for predicting outcomes.

**Table 2:** The relationship between the difference in ISCU expression and clinical characteristics.

	ISCU-High	ISCU-Low	P-value
	(N=15)	(N=25)	
**ISCU.Exp**			**1.70E-11**
Mean (SD)	7.60 (1.55)	2.08 (2.04)	
Median [Min, Max]	8.00 [6.00, 12.00]	4.00 [0.00, 4.00]	
Age			0.369
Mean (SD)	48.1 (8.67)	50.9 (10.10)	
Median [Min, Max]	51.0 [35.0, 66.0]	52.0 [31.0, 73.0]	
**FIGO**			**0.0004**
I	11 (73.3%)	4 (16.0%)	
II	4 (26.7%)	9 (36.0%)	
III	0 (0%)	12 (48.0%)	

Bold emphasis shows the results of analyses with statistical differences.

## Discussion

The occurrence and treatment of cervical cancer is a complex pathological process involving many aspects, among which mitochondrial metabolic reprogramming as a significant differential pattern of tumors has received extensive attention (17). Mitochondria are vital bioenergetic and biosynthetic factories that are crucial for normal cellular function and human health (18). Alterations in metabolic pathways, including mitochondrial DNA mutations, TCA cycle enzyme defects, mitochondrial oxidative phosphorylation deficiencies, oxidative stress, abnormal oncogenes, and tumor suppressor signal transduction, disrupt redox balance, leading to mitochondrial dysfunction and promoting cancer development (19–22). In addition, the number of mitochondria is significantly reduced in certain cancer cells, and the morphology of mitochondria becomes more spherical, which can affect mitochondrial function (23). Tumor cells actively participate in glycolysis and its branching pathways as well as TCA cycle metabolism, utilizing metabolic reprogramming to generate ATP, NADPH, and macromolecules for cancer cell growth and development (21). Initial progress has been made in immunotherapy for cervical cancer, but there are still many patients who do not benefit from it. Therefore, it is still important to explore new immune therapy evaluation systems and novel biomarkers. We started with mitochondria-related metabolic differential genes in cervical cancer and constructed a prognosis index to evaluate the prognosis and immune therapy sensitivity of cervical cancer patients. We found significant differences in prognosis among patients with different scores and variations in immune therapy effectiveness. These analysis results indicate that changes in mitochondrial structure and function are important features of cervical cancer and potential biological targets for its treatment. Alterations in mitochondrial metabolic patterns are also one of the important indicators for clinical prognosis evaluation.

In this study, 1123 differentially expressed genes related to metabolic pathways were screened using the TCGA database and GSE52904. Subsequently, we performed univariate COX regression analysis and LASSO COX regression analysis, combined with clinical indicators of cervical cancer patients, to identify 5 key genes. We used these genes to construct a risk index for evaluating patient prognosis. Patients were classified into high-risk and low-risk groups based on the prognosis index. The relationship between the prognosis index and immune infiltration, as well as immune checkpoint inhibitor therapy, was determined through immune analysis. Due to the survival time of patients in the validation set GSE52904 being less than 3 years, we chose 0.5, 1, and 2 years as evaluation time points to assess the predictive ability. In the training set and validation set, the AUC for 1-year survival was 0.81 and 0.80, respectively, indicating good predictive ability of the model. Patient stratification based on the prognosis index revealed significant differences in the proportions of immune infiltrating cells and the expression of immune checkpoint inhibitor-related genes, which can effectively distinguish individuals sensitive to immune therapy. Finally, combining the analysis results with a literature review, we found that the Iron-Sulfur Cluster Assembly Enzyme (ISCU) gene has good predictive value in the prognosis and diagnosis of cervical cancer. Therefore, we collected cervical cancer patient tissues from XiJing Hospital and conducted immunohistochemical analysis, revealing a significant negative correlation between its expression and clinical stage (24).

Analysis results revealed that the prognostic index of mitochondrial metabolism-related genes can influence the composition of immune cells in the tumor microenvironment, and patients with different risk prognostic indexes exhibit significant differences in cellular components within the microenvironment. Compared to normal tissue, T lymphocytes, B lymphocytes, and macrophages are more abundant in normal tissue, while their proportions are significantly reduced in tumor tissue, revealing the immune characteristics of cervical cancer. Our scoring revealed that patients with a high prognostic index have a higher proportion of plasma cells, CD8+ T cells, immature CD4+ T cells, Treg cells, while patients with a low prognostic index have a higher proportion of three types of macrophages. T and B lymphocytes play a significant role in humoral and cellular immunity. The differences between high and low-risk score patients indicate that high-risk score patients may benefit more from immunotherapy. However, a high accumulation of Treg cells is observed in patients with a high index, and it has been reported that a greater accumulation of Treg cells in tumors leads to a worse prognosis (25). Based on our scoring, many late-stage and recurrent patients were found to have a high-risk score, and their poor prognosis may be associated with a significant infiltration of Treg cells. We speculate that this result may be due to the complex regulatory mechanisms among T, B, and Treg cells. However, overall, patients with a high index of cervical cancer have a poor prognosis. Current research has found that macrophages play a significant role in eliminating tumor cells and regulating tumor immunity (26). Patients with a low index may have a better clinical prognosis, which could be associated with the accumulation of macrophages in tumors. Tumor cells adapt to the increasing energy and biosynthetic demands by reprogramming relevant metabolic pathways (27). In the tumor microenvironment, tumor development-driven nutrient depletion and excessive production of metabolic byproducts regulate the metabolic reprogramming of tumor-infiltrating immune cells and activate signaling pathways to control the polarization of different types of immune cells. This induces the loss of anti-tumor immune response mediated by metabolic abnormalities and contributes to the establishment of an immune-suppressive tumor microenvironment (28, 29). Mitochondria, as cell organelles with diverse biological functions and high plasticity, play a crucial role in regulating metabolism and activating immune cells (30, 31). Studies have shown that mitochondrial dysfunction in various cells within the tumor microenvironment, including tumor cells and immune cells, is a significant factor in the occurrence, development, and metastasis of cancer (32). Therefore, starting from the role of mitochondrial energy metabolism in regulating tumor immunity, we screened genes associated with the prognosis of cervical cancer patients and constructed a risk score for prognosis. Our results also demonstrated stability in the training set, providing a novel explanation for evaluating the prognosis of cervical cancer patients at the level of mitochondrial energy metabolism genes. We identified five key genes from the metabolic-related gene set of cervical cancer.

Mitochondrial energy metabolism and biosynthesis play a crucial regulatory role in the activation of immune cells, while tumor cells competitively consume glucose and the tumor hypoxic microenvironment leads to mitochondrial damage and excessive generation of reactive oxygen species, resulting in immune cells being in a state of long-term metabolic insufficiency and high oxidative stress environment, which disrupts the activation of immune cells and their tumor immune surveillance function, thereby acquiring tumor immune evasion ability (33–35). Therefore, mitochondrial metabolism plays a crucial physiological role in reshaping the tumor microenvironment and influencing tumor immune therapy. Immune checkpoint inhibitors have been used in advanced and recurrent cervical cancer, but there is considerable individual variation in the effectiveness of immune therapy. Therefore, we analyzed whether differential genes related to cervical cancer mitochondrial metabolism can predict the effectiveness of immune therapy in patients. TIDE analysis found that the scores of low-index patients were significantly higher than those of high-index patients, indicating that the immune therapeutic effect may be unsatisfactory in low-index patients. TMB, as a predictive factor for the therapeutic response of tumor immune checkpoint inhibitors, is associated with better prognosis in patients with high TMB when receiving PD-(L)1-based therapy (36, 37). The poor immune therapeutic effect in low-index patients may be due to their lower TMB scores. Analysis of the relationship between prognostic index-related genes and genes regulating immune checkpoint inhibitors revealed a correlation between the ISCU gene and PD-1, PDL-1, CTLA4, suggesting that the predictive role of risk scoring may be related to immune checkpoint inhibitor-related genes. Drug sensitivity analysis revealed that low-index patients exhibited significantly higher sensitivity to mitochondrial inhibitors, insulin-like growth factor 1 receptor (IGF-1R) and insulin receptor (IR) inhibitors, IRAK4, demonstrating better drug sensitivity.

Moreover, we observed a significant positive correlation between ISCU and immune checkpoint inhibitor-regulating genes (PD1, PDL1, CTLA4, etc.) among the core genes, which prompted us to investigate its role and potential mechanisms in cervical cancer immune therapy and regulation of the immune microenvironment. ISCU, as an iron assembly enzyme, plays an important role in regulating cellular iron metabolism (38). Studies have found that interfering with its expression can lead to increased intracellular reactive oxygen species (ROS), and the impact of iron metabolism on intracellular ROS has been well established (39). Our study revealed a decrease in ISCU expression with increasing stage and a negative correlation with prognosis. Through literature review, it is crucial to have an excess of intracellular reactive oxygen species (ROS) in the immune microenvironment. Many immune cells in the microenvironment, such as macrophages, dendritic cells, T cells, are regulated by ROS, and tumor treatment methods targeting ROS have shown promising results in animal experiments (40, 41). Our analysis results revealed that the prognostic index can effectively distinguish individuals who are sensitive to immune checkpoint inhibitor therapy. It is hypothesized that in cervical cancer, ISCU may regulate the function of immune cells in the tumor microenvironment by reprogramming intracellular iron metabolism to affect ROS, thereby influencing the tumor immune microenvironment and the effectiveness of immune therapy.

Our analysis exhibits several significant characteristics compared to earlier publications. Firstly, we have acknowledged the potential involvement of metabolism-related genes in regulating the therapeutic immune microenvironment, and by developing a prognostic index, we have offered patients more accurate stratification and treatment options. Secondly, the construction of the prognostic index did not solely focus on genes influencing prognosis, but instead, we integrated clinical information by combining it with genes, proposing a novel approach to predict prognosis and immunotherapy. Thirdly, based on the analysis results, we identified sensitive drugs for patients with different risks, where patients with high index scores exhibited good responsiveness to immunotherapy, while patients with low scores showed significant response to kinase inhibitors. Our analysis demonstrates that utilizing mitochondrial metabolism-related genes for the classification of cervical cancer patients can effectively differentiate distinct prognostic groups. Additionally, when combined with TIDE and TMB analysis, the prognostic index can assess the immunotherapeutic effectiveness in patients with varying scores. Based on a series of analyses, ISCU has demonstrated potential clinical predictive value, exhibiting a strong correlation with immune checkpoint-related genes, and may serve as a potential biomarker for immunotherapy in cervical cancer. However, the current analysis still has several limitations. Firstly, the value of prognostic scoring still requires further validation with a large number of clinical samples. Secondly, the evaluation of immunotherapeutic effectiveness still needs to be explored at the genetic, RNA, and protein levels. Additionally, the molecular mechanisms of ISCU gene occurrence in cervical cancer also need to be elucidated through further basic experiments.

## Conclusion

Utilizing the TCGA database, we investigated mitochondrial metabolism-related differentially expressed genes associated with cervical cancer and developed a prognostic index by analyzing these genes. The prognostic indicator was successful in assessing the outlook of patients with cervical cancer, uncovering unique clinical results within various clinical categories. Additionally, a connection was noted between the risk assessment and the infiltration of immune cells in tumor tissue, along with immune-related functions in patients with tumors. By combining TIDE and TMB scores, we successfully assessed the immunotherapy response in patients. The high-index group showed increased TMB, greater immune cell infiltration, and lower TIDE scores, indicating a possible advantage from immunotherapy. Conversely, the low-index group displayed increased sensitivity to metabolism-related antitumor agents, particularly multiple kinase inhibitors. To summarize, we cautiously pinpoint ISCU as a potential indicator in cancer treatment.

## Data availability statement

The datasets presented in this study can be found in online repositories. The names of the repository/repositories and accession number(s) can be found in the article/**Supplementary Material**.

## Ethics statement

The studies involving humans were approved by Medical Ethics Committee of the First Affiliated Hospital of the Air Force Medical University. The studies were conducted in accordance with the local legislation and institutional requirements. The participants provided their written informed consent to participate in this study.

## Author contributions

BM: Data curation, Formal analysis, Visualization, Writing – original draft, Writing – review & editing. CR: Writing – review & editing. YY: Writing – review & editing. SZ: Writing – review & editing. JL: Writing – review & editing, Supervision. HY: Writing – review & editing, Funding acquisition, Supervision.
