# Supplementary material for: Immune cell infiltration and prognostic index in cervical cancer: insights from metabolism-related differential genes
Source: Front Immunol. 2024 May 22;15:1411132. doi: 10.3389/fimmu.2024.1411132 (PMC11150690; doi:10.3389/fimmu.2024.1411132)
Supplement: Supplementary file 1 [file DataSheet_1.docx]

Supplementary Material

##
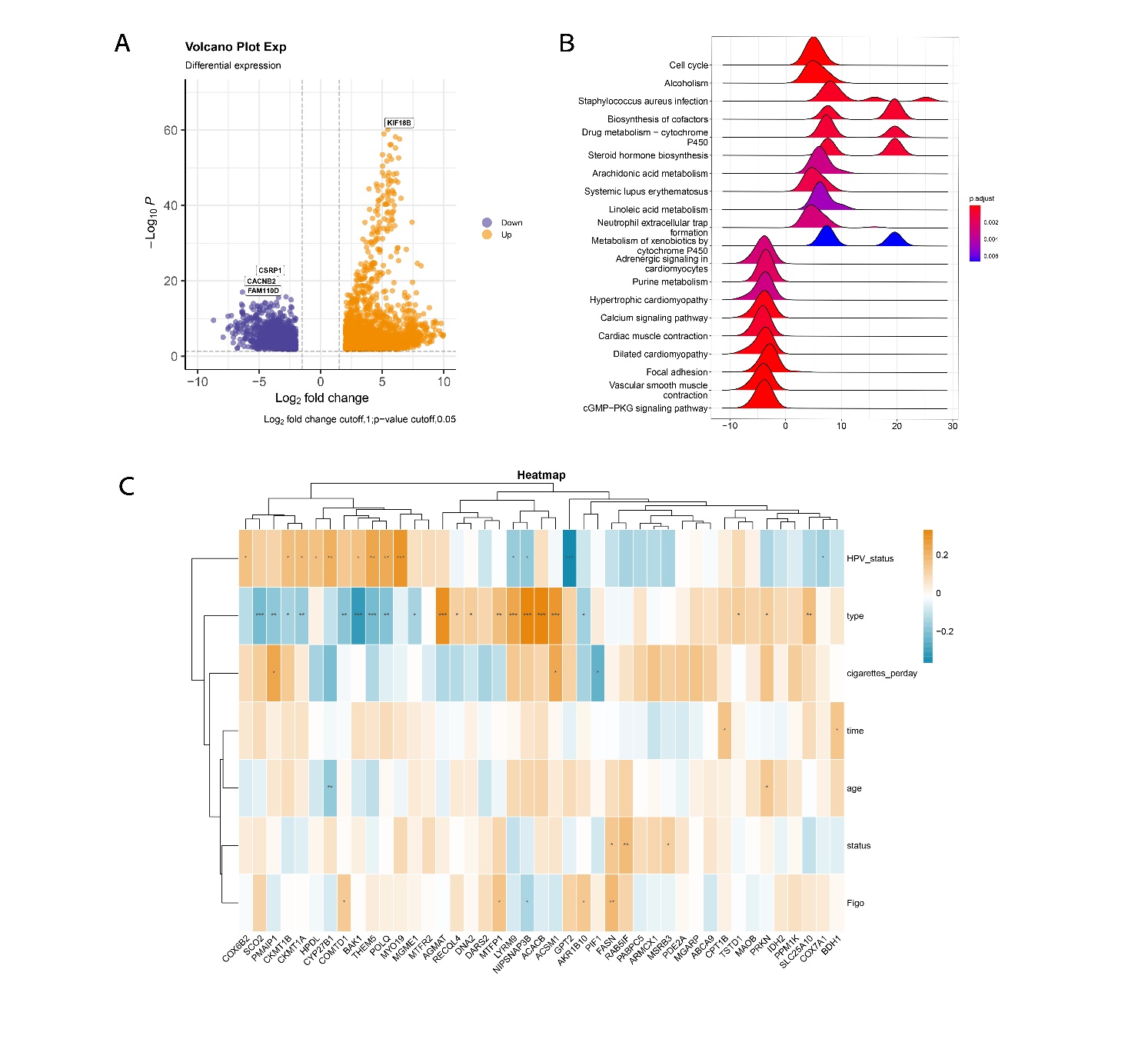
Supplementary Figures

**Supplementary Figure 1.**

Analysis of metabolic differential gene expression, biological processes, and clinical correlations.(A) Metabolic differences in cervical cancer gene volcano map; orange is up-regulated genes in tumors, purple is down-regulated genes in tumors.(B) Differential gene GSEA biological process enrichment analysis.(C) Correlation between metabolic differential genes and clinical information (the top 50 genes with the most significant differences were selected).(P values are shown as: *P < 0.05; **P < 0.01; ***P < 0.001)


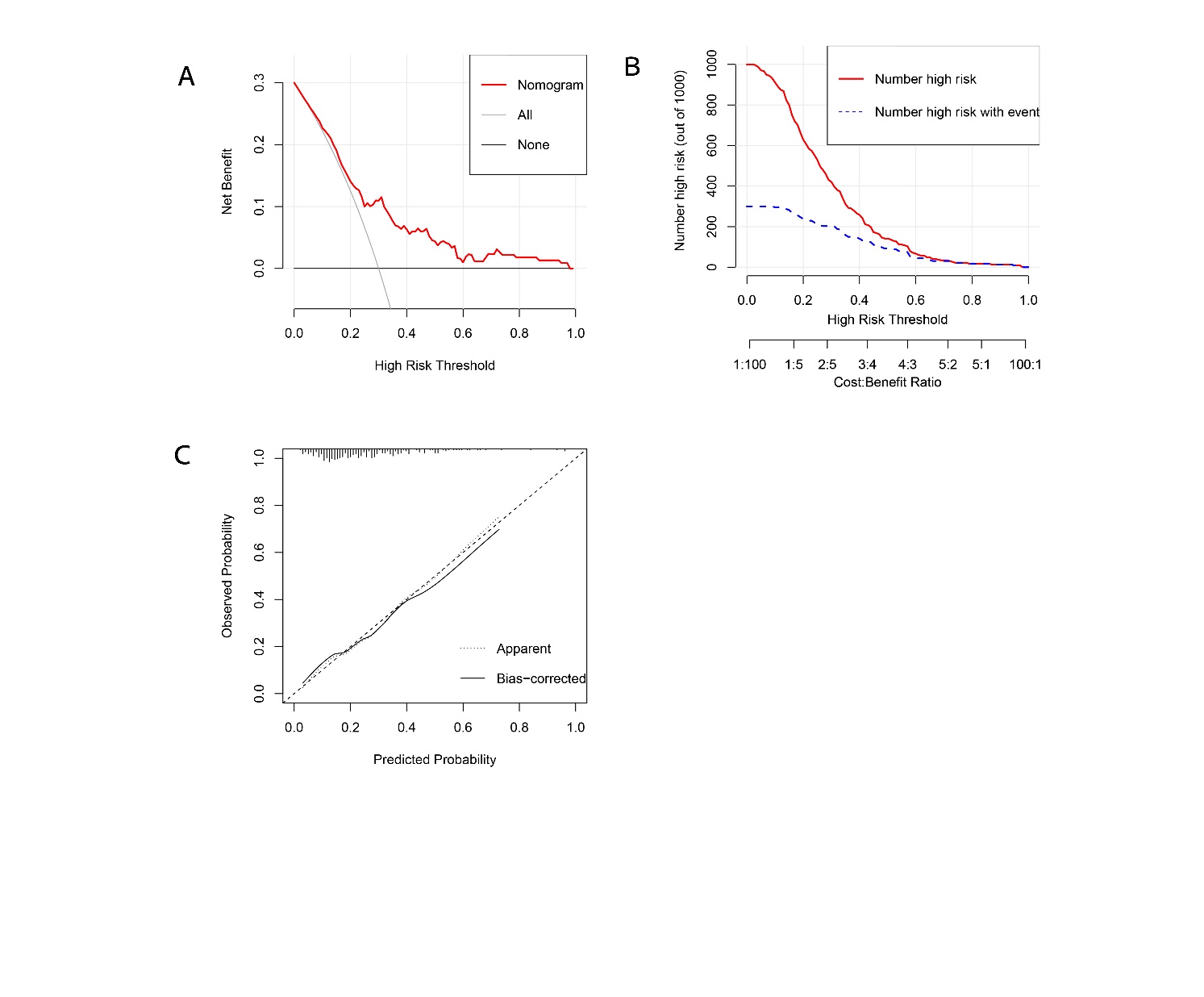


**Supplementary Figure 2.**

Prognostic score effect evaluations. (A) Clinical decision curve curves for prognostic scores in the TCGA database. (B) Clinical impact curves for prognostic scoring in the TCGA database. (C) Calibration curves for prognostic scores in the TCGA database.


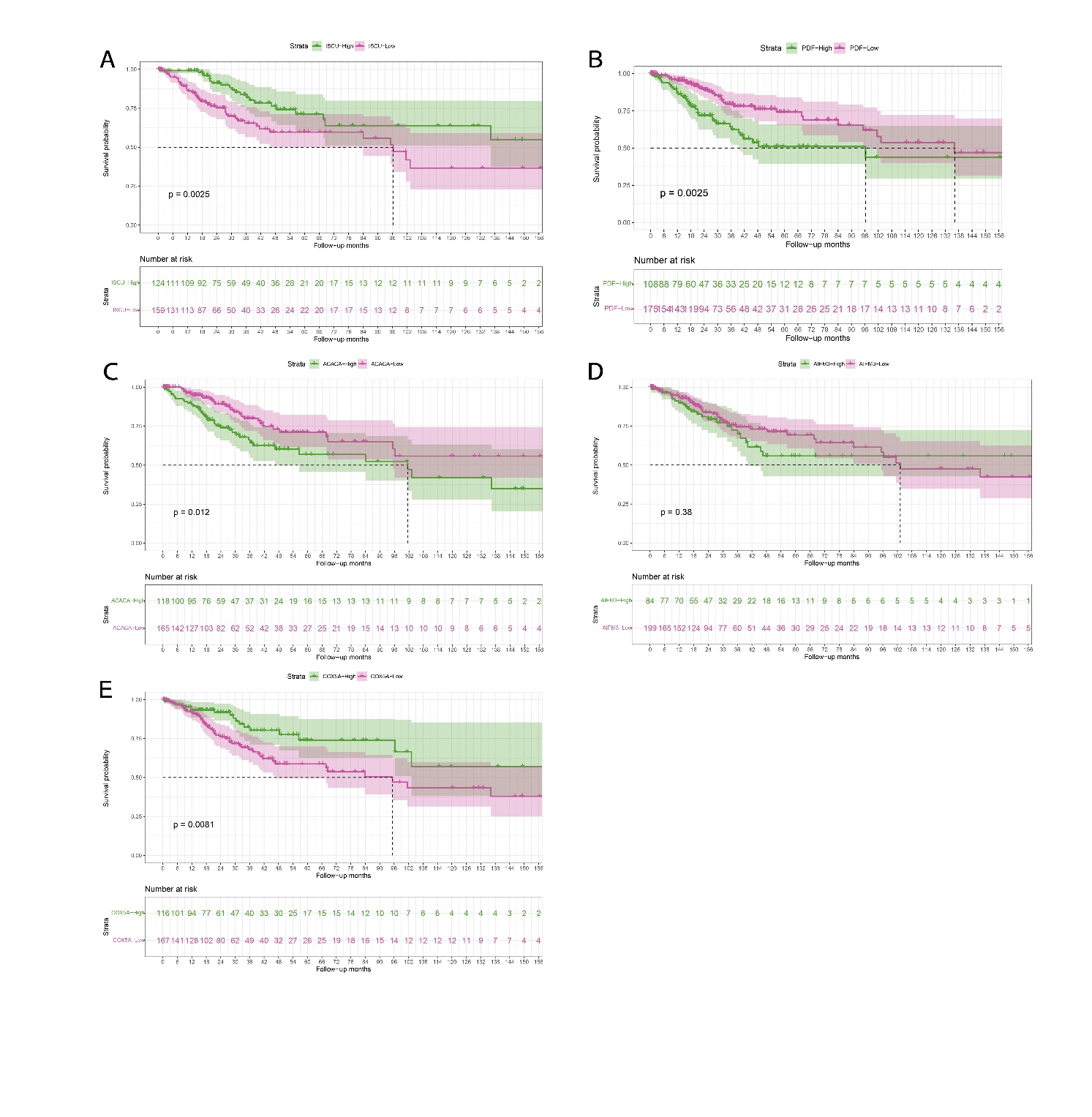


**Supplementary Figure 3.**

Survival analysis of (A) ISCU, (B) PDF, (C) ACACA, (D) AIFM3, and (E) COX5A in the TCGA database.


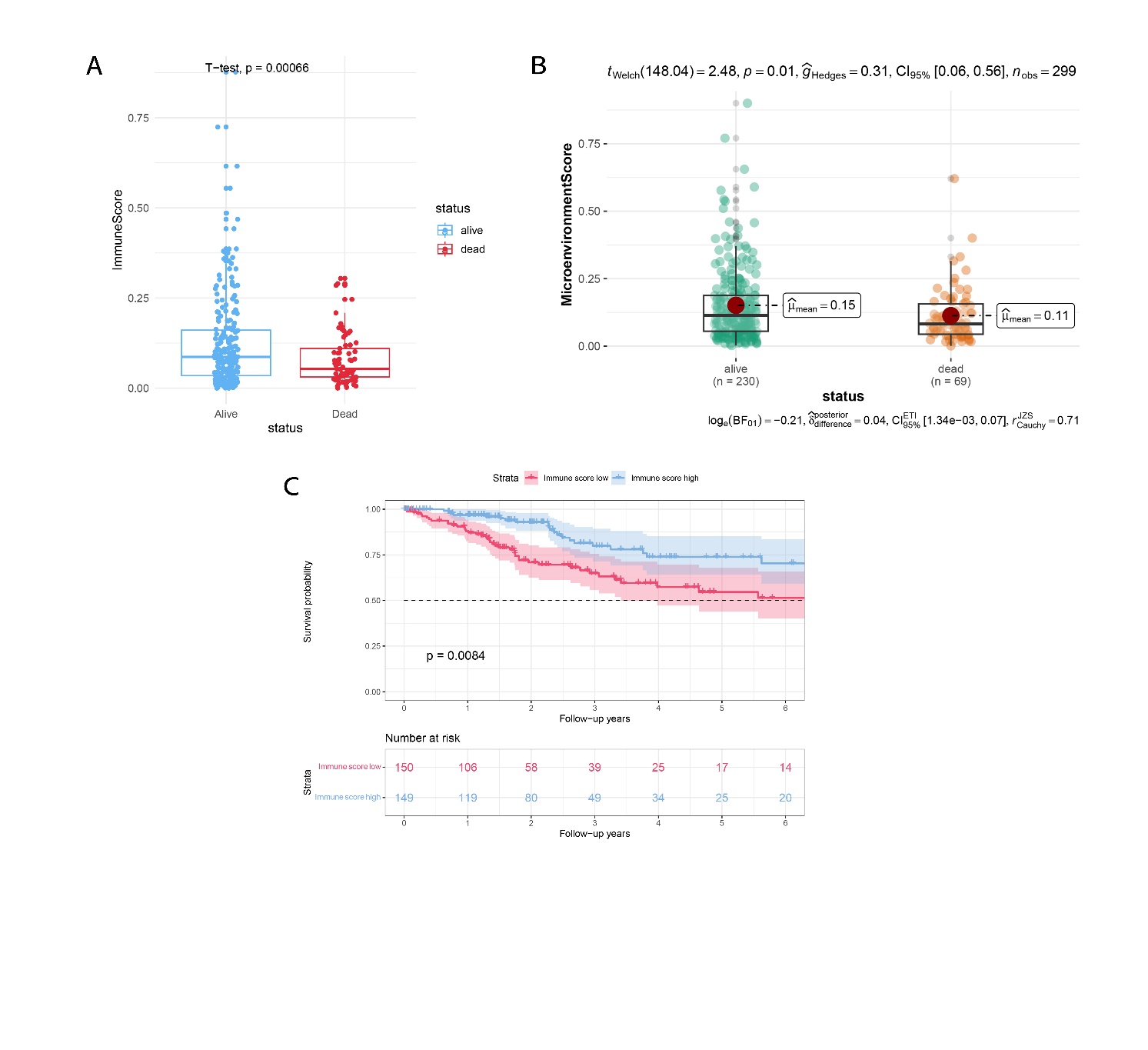


**Supplementary Figure 4**.

ImmuneScore difference analysis of surviving and deceased cervical cancer patients.(A) ImmuneScore differences between surviving and deceased patients in the TCGA database.(B) Difference in MicroenvironmentScore between surviving and deceased patients in the TCGA database.(C) Survival curves of surviving versus deceased patients in the TCGA database.


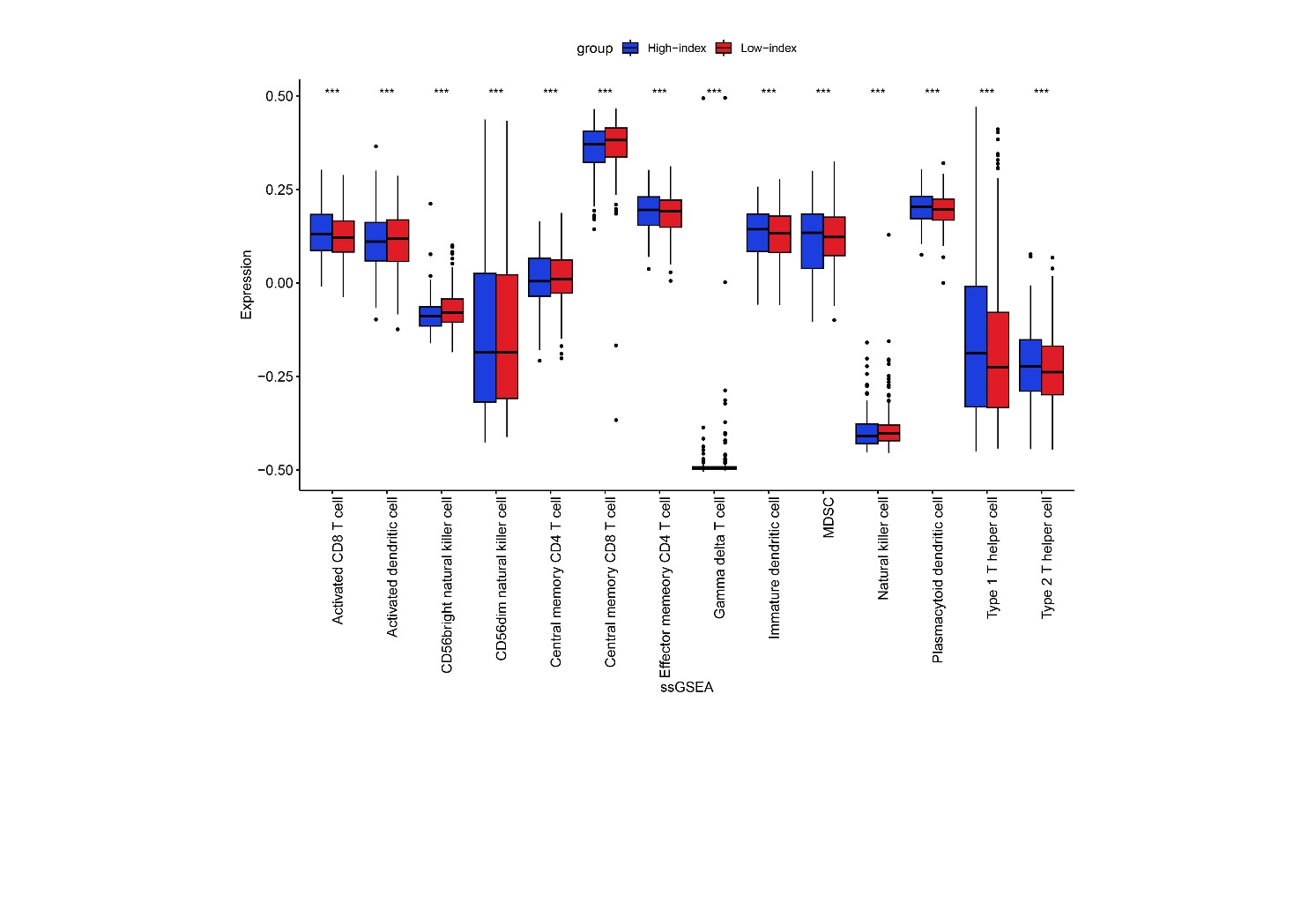


**Supplementary Figure S5.** Analysis of the immune profile in specific subgroups using ssGSEA scores.

(P values are shown as: *P < 0.05; **P < 0.01; ***P < 0.001).


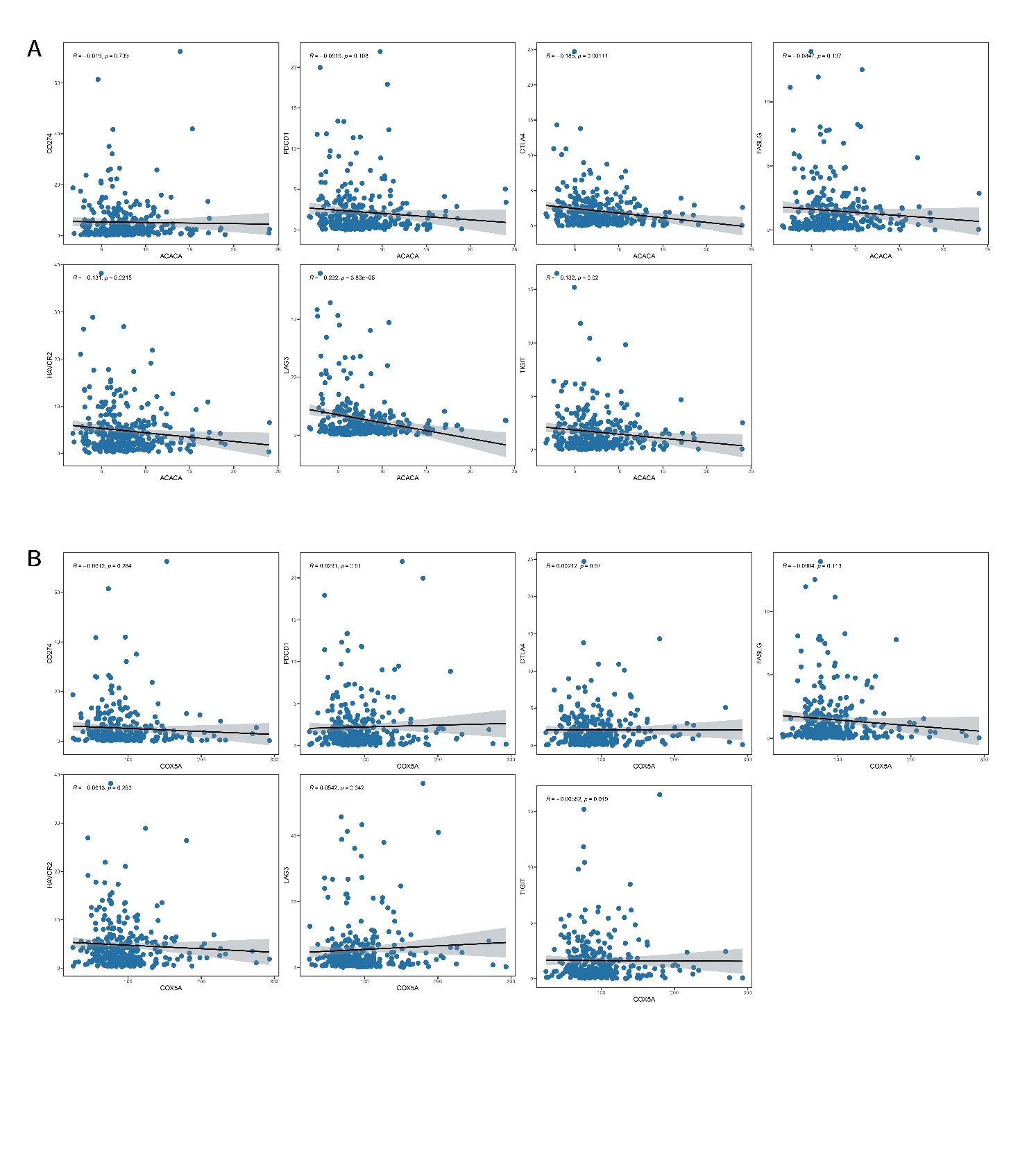
Supplementary Figure S6. Key genes correlate with immune checkpoint inhibitor genes(CD274,PDCD1,CTLA4,FASLG,HAVCR2,LAG3,TIGIT).(A) ACACA and immune checkpoint inhibitor gene correlation.(B) COX5A correlates with immune checkpoint inhibitor genes.ACACA: Acetyl-CoA Carboxylase Alpha；COX5A: Cytochrome C Oxidase Subunit 5A

**
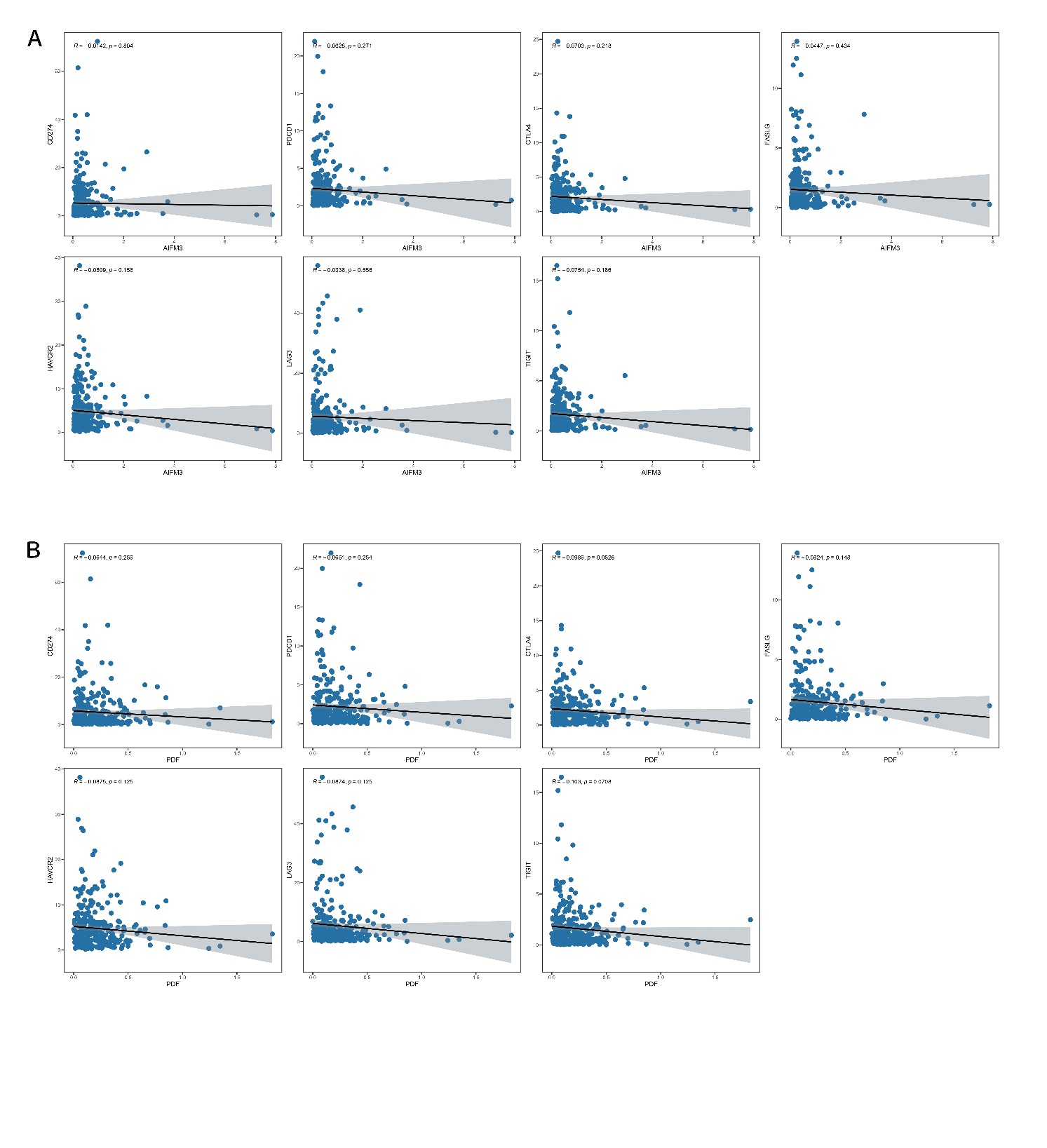
Supplementary Figure S7.** Key genes correlate with immune checkpoint inhibitor genes(CD274,PDCD1,CTLA4,FASLG,HAVCR2,LAG3,TIGIT).(A) AIFM3 correlates with immune checkpoint inhibitor genes.(B) PDF correlates with immune checkpoint inhibitor genes.AIFM3 : Apoptosis Inducing Factor Mitochondria Associated 3； PDF : Peptide Deformylase, Mitochondrial


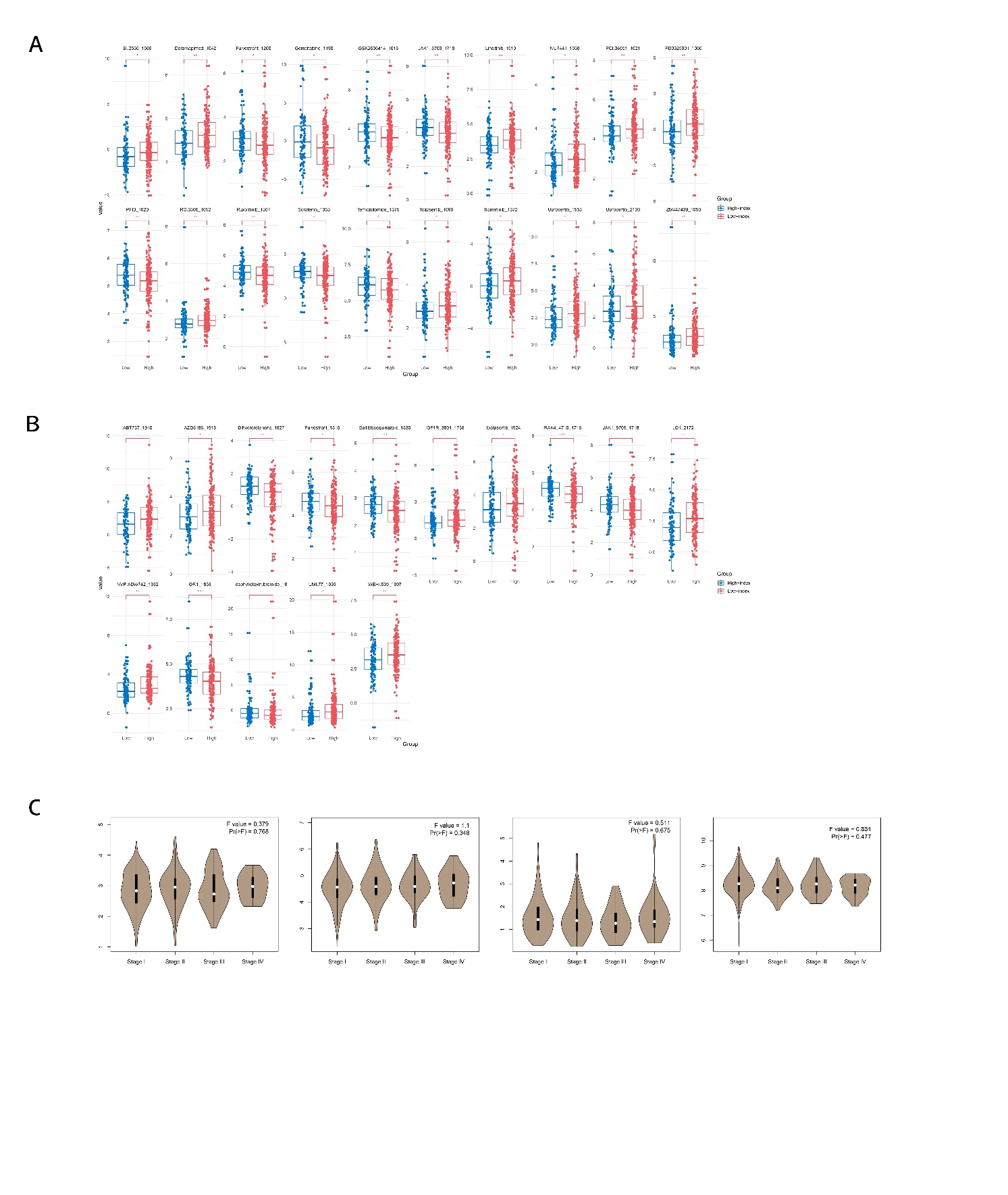


**Supplementary Figure S8．** (A) (B) OncoPredict analysis of differences in drug sensitivity between high and low index groups.(C) Expression of PDF,ACACA ,AIFM3 ,COX5A in cervical cancers of different stages.

(P values are shown as: *P < 0.05; **P < 0.01; ***P < 0.001).
